# Effects of dietary proanthocyanidin supplementation on growth performance, immune function, antioxidant capacity, and gut microbiota in weaned pigs

**DOI:** 10.3389/fmicb.2025.1704019

**Published:** 2026-01-14

**Authors:** Yong Qiao, Jiahao Liu, Kunhong Xie, Bing Yu, Yuheng Luo, Ping Zheng, Xiangbing Mao, Hui Yan, Jun He

**Affiliations:** 1 Animal Nutrition Research Institute, Sichuan Agricultural University, Chengdu, Sichuan, China; 2 Key Laboratory of Animal Disease-Resistance Nutrition, Chengdu, Sichuan, China

**Keywords:** PRO, growth performance, nutrient digestibility, immune function, gut microbiota

## Abstract

**Introduction:**

Proanthocyanidin (PRO), a widely consumed type of dietary polyphenolic compound, exhibits diverse health-promoting properties due to its structure rich in abundant hydroxyl groups. However, the effects of dietary PRO supplementation on growth performance, immune function, antioxidant capacity, and gut microbiota in weaned piglets remain unexplored.

**Methods:**

In this study, 800 hybrid barrows of Duroc × Landrace × Yorkshire (DLY) piglets, aged 28 days and with an average body weight of 9.40 ± 0.14 kg, were randomly assigned to five groups. Each group of piglets was continuously administered one of the following five dietary treatments: a basal diet (control group) or the basal diet supplemented with PRO at different doses of 15 mg, 30 mg, 60 mg, or 120 mg per kilogram of feed for 28 days.

**Results:**

On day 29, dietary PRO treatment showed a dose-dependent improvement in average daily gain (ADG, linear, *p* = 0.042), average daily feed intake (ADFI, linear, *p* = 0.078), and the digestibility of nutrients, including dry matter (DM), crude protein (CP), crude fat (EE), and gross energy (GE) (*p* < 0.05). Compared to the control group, PRO supplementation linearly reduced (*p* < 0.05) the concentrations of proinflammatory cytokines such as tumor necrosis factor alpha (TNF-α), interleukin-1 beta (IL-1β), and interleukin 6 (IL-6). In contrast, the levels of serum immunoglobulins, such as IgG, IgA, and IgM, as well as the activities of GSH and T-AOC, were linearly elevated (*p* < 0.05) by PRO supplementation in the piglet diet. In addition, dietary supplementation with 30 mg/kg PRO not only increased the abundance of butyrateproducing bacteria, such as *Fournierella*, *Oscillospira*, *NK4A214_group*, and *UCG-005*, at the species level but also tended to elevate (*p* < 0.1) the concentration of butyrate in the rectum.

**Conclusions:**

These results suggest that PRO-containing feed might be a potential dietary strategy for improving gut homeostasis and overall health in weaned pigs.

## Background

Proanthocyanidin (PRO) is ubiquitous in various parts of plants, including seeds, leaves, flowers, skins, and shells. PRO is formed by the polymerization of flavan-3-ol monomers through C–C and C–O–C bonds, featuring a unique C6–C3–C6 flavonoid backbone (Yang et al., 2021; Jonker and Yu, 2017). Generally, PRO can be classified into oligomeric PRO (OPC; 2–4 monomer units) or polymeric PRO (PPC; ≥5 units) based on its polymerization degree (Nie et al., 2023). Due to their structural characteristics formed through condensation reactions, PROs are also classified as condensed tannins (Quideau et al., 2011). Particularly, the copious existence of phenyl and hydroxyl groups within molecular structures may be crucial for the exceptional antioxidant capability of PRO (Jin et al., 2020). Indeed, PRO exhibits a stronger antioxidant capability than vitamin C or vitamin E, and this has been confirmed by an *in vitro* experiment (Shi et al., 2004). Furthermore, previous studies have reported that PRO exerts effective free radical scavenging activity by bolstering Nrf/ARE pathway expression and inhibiting NADPH oxidase, thereby exhibiting multifaceted antioxidant defense potential (Yvonne et al., 2008; Su et al., 2018; Abdur et al., 2019).

In addition to the well-known antioxidant capacity, PRO has garnered extensive research attention for its additional beneficial effects, including antimicrobial, anti-inflammatory, anticancer, and anti-aging effects (Abdur et al., 2019; Gopalakrishnan et al., 2018; Xie et al., 2023; Nie and Stürzenbaum, 2019). For example, PRO inhibited the activation of M1 macrophages by regulating the NF-κB signal pathway in rats after lipopolysaccharide (LPS) injection (Shi et al., 2023). Similarly, another *in vitro* study demonstrated that PRO markedly decreased the production of reactive oxygen species (ROS) and cytokines such as interleukin-1 beta (IL-1β), interleukin 8 (IL-8), and tumor necrosis factor alpha (TNF-α), as well as the enzymes elastase-2 and MMP-9 in LPS-stimulated human neutrophils (Michel et al., 2019). It has been reported that PRO has the characteristic of enhancing CD8^+^ cytotoxic T lymphocyte activity, as shown by increases in the expression of co-stimulatory molecules (e.g., CD80, CD86, MHC-I, and MHC-II) on the cell surface (Zhang et al., 2017). Previous studies have shown that PRO can maintain intestinal health by up-regulating the expression of *ZO-1*, *claudin-1*, and *occludin-1* related to tight junctions and special intestinal flora such as *Limosilactobacillus reuteri* and *Akkermansia* (Liu and Bolling, 2024; Andersen-Civil et al., 2022).

While PRO has demonstrated health-promoting effects in diverse biological systems, its application in swine feed formulations and its impact on gut health and growth performance in weaned piglets remain largely unexplored. Given PRO’s potent beneficial effects, we hypothesize that it can improve production performance in pigs by enhancing their antioxidant capacity and gut health. Therefore, the objectives of this study were to evaluate the effects of dietary PRO supplementation on growth performance in weaned pigs, determine if PRO can mitigate oxidative stress, inflammatory responses, and intestinal flora disturbances caused by weaning stress, and explore the potential applications of PRO in pig production.

## Materials and methods

The animal experiment in this study was carried out with approval from the Animal Care and Use Committee of Sichuan Agricultural University (Chengdu, China). PRO (Cat No. DAT00140, purity ≥95%) was purchased from Guilin Fengpeng Biological Technology Co., Ltd. (Guangxi, China).

### Experimental design, diet, and animal housing

A total of 800 28-day-old pigs (Large White × Landrace × Duroc, average body weight 9.40 ± 0.14 kg) were randomly divided into five groups (four pens in each group, 40 pigs in each pen) and fed diets containing varying PRO concentrations (0, 15, 30, 60, and 120 mg/kg) for 28 days. The diet was formulated (Specific parameters were required to keep confidential by the company) based on the National Research Council (2012). The pigs were raised in ventilated barns with slatted floors (4 m × 5 m) under appropriate temperature (25 ± 2 °C) and humidity (60 ± 3%), and they were provided with ample access to food via two strip-shaped feed troughs and water through five conveniently placed water nipples.

### Sample collection

During the last 3 days of the experiment (from 21:00 on day 25 to 21:00 on day 28), fresh fecal samples were collected from the piglet with BW closest to the average in each pen (*n* = 4) using rectal grabs with valve bags. After collection, fecal nitrogen was fixed with 10% H_2_SO_4_ (100 mL/kg) for the determination of apparent total tract nutrient digestibility. On day 28, freshly expelled feces from the pigs were immediately collected into sterile frozen tubes and stored at −80 °C for 16S analysis. On day 29, after 12 h of fasting, the piglets from which fecal samples had been collected were used for blood collection. Blood samples were drawn from the precaval vein into 20 mL plain blood collection tubes, and upper serum was obtained by centrifugation at 3,500 × g for 15 min (4 °C).

### Growth performance

Body weight on days 1 and 29 and average daily feed intake (ADFI) were recorded during the experiment. Average daily gain (ADG), average daily feed intake (ADFI), and the feed-to-gain ratio (F:G) were calculated based on the above data.

### Apparent total tract nutrient digestibility analysis

Fecal and diet samples stored at −20 °C were thawed, homogenized, and dried at 65 °C until achieving a consistent weight (with consecutive weighing errors ≤ 0.5 g) prior to nutrient digestibility analysis, and hydrochloric acid-insoluble ash was used as an endogenous indicator. The measurements of dry matter (DM), crude protein (CP), EE, and ash were according to the standard methods of the Association of Official Analytical Chemists (AOAC, 2007), while gross energy (GE) was measured using an adiabatic bomb calorimeter.

### Serum biochemical analysis

Serum antioxidant capacities, such as catalase (CAT), malondialdehyde (MDA), glutathione (GSH), total superoxide dismutase (T-SOD), total antioxidant capacity (T-AOC), hydroxyl free radical (•OH), superoxide anion (O^2−^), and metabolites, such as glucose (GLU), triglyceride (TG), and total cholesterol (TC), were measured using assay kits purchased from Nanjing Jiancheng Biotechnology Co., Ltd. (Jiangsu, China). Assay kits for serum inflammatory cytokines, such as tumor necrosis factor alpha (TNF-α), interleukin-1 beta (IL-1β), and interleukin 6 (IL-6), as well as immunoglobulins such as immunoglobulin A, G, and M (IgA, IgG, IgM), were purchased from Jiancheng Enzyme-linked Biotechnology Co., Ltd. (Jiangsu, China). All procedures were performed according to the manufacturer’s instructions.

### Rectal microbiota and SCFA analysis

Genomic DNA was extracted from fecal samples stored in an ultra-low temperature freezer at −80 °C according to the method described previously. The primers 341F and 806R were employed to amplify the V3–V4 regions of the 16S rRNA. Sequencing was performed using Beijing Novogene Biotech’s QIIME2 platform (Beijing, China), with analysis based on amplicon sequence variants (ASVs). Raw data and analysis procedures are available on the NovoMagic platform under project number X101SC23090186-Z01-J001. Microbial community diversity and richness were represented by alpha diversity indexes, such as Chao1, Observed_features, Dominance, Shannon, Simpson, and PIEOU_e, as well as beta diversity indexes such as principal coordinate analysis (PCOA), principal component analysis (PCA), and non-metric multidimensional scaling (NMDS). The predominant microbiota within each group are showcased through an LEfSe plot, utilizing a screening threshold of a linear discriminant analysis (LDA) score greater than 4. The concentrations of short-chain fatty acids (SCFAs) in the fecal samples were measured using a gas chromatography system (Varian CP-3800, Agilent Technologies, Santa Clara, CA, United States).

### Statistical analysis

All results were expressed as means ± standard error and subjected to one-way analysis of variance (ANOVA) with SPSS 27.0 (IBM, Chicago, IL, United States). Polynomial multiple comparisons (linear and quadratic) were performed using curve estimation to assess the effect of PRO dose. A *p*-value of <0.05 was considered statistically significant, while a *p*-value between 0.05 and 0.1 was interpreted as a trend.

## Results

### Growth performance

Table 1 reveals the effects of dietary PRO supplementation on growth performance in weaned pigs. The ADG increased significantly with rising PRO content and reached a plateau in the 30 mg/kg PRO group (linear, *p* = 0.042; quadratic, *p* = 0.055). Meanwhile, the ADFI showed a linear increasing trend with increasing levels of PRO (linear, *p* = 0.078). However, there were no differences in FBW and F:G between the PRO and the CON groups.

**Table 1:** Effect of PRO supplementation on growth performance in the weaned pigs^1^.

Items^2^	PRO (mg/kg)					SEM	*p*-value		
	0	15	30	60	120		ANOVA	Linear	Quadratic
IBW (kg)	9.34	9.45	9.38	9.44	9.41	0.14	0.99		
FBW (kg)	27.62	28.90	29.61	29.18	29.37	0.36	0.478	0.142	0.168
ADG (g/d)	652.76^b^	684.42^a,b^	722.10^a^	705.15^a^	713.07^a^	9.30	0.136	0.042	0.055
ADFI (g/d)	907.67	953.95	986.93	981.17	998.91	16.64	0.472	0.078	0.169
F:G	1.39	1.38	1.37	1.39	1.40	0.05	0.924	0.648	0.674

IBW, initial body weight; FBW, final body weight; ADFI, average daily feed intake; ADG, average daily gain; F:G, feed:gain ratio.

1 Mean and total SEM are listed in separate columns, *N* = 4.

2 ^a,b,c^Mean values within a row with different superscript letters differ significantly, *p* < 0.05. Abbreviations and statistical notation are consistent across all tables and figures.

### Nutrient digestibility

As shown in Table 2, dietary PRO supplementation significantly increased the apparent total tract digestibility of DM (linear: *p* = 0.001; quadratic: *p* = 0.001), EE (linear: *p* = 0.006; quadratic: *p* = 0.015), GE (linear: *p* = 0.001; quadratic: *p* = 0.001), CP (linear: *p* = 0.02; quadratic: *p* = 0.01), ASH (linear: *p* = 0.002; quadratic: *p* = 0.004) with increasing levels of PRO in the diet of the weaned pigs.

**Table 2:** Effect of PRO supplementation on nutrient digestibility in the weaned pigs.

Items	PRO (mg/kg)					SEM	*p*-value		
	0	15	30	60	120		ANOVA	Linear	Quadratic
DM%	87.61^b^	88.46^a^	88.89^a^	88.94^a^	89.09^a^	0.002	0.004	0.001	0.001
EE%	86.63^b^	87.44^a,b^	87.74^a^	87.77^a^	88.07^a^	0.002	0.079	0.006	0.015
GE%	86.73^b^	87.81^a^	88.42^a^	88.42^a^	88.39^a^	0.002	0.002	0.001	0.001
CP%	78.39^b^	79.50^a^	81.11^a^	80.72^a^	80.44^a^	0.003	0.051	0.020	0.010
ASH%	54.54^b^	55.99^a,b^	56.80^a^	57.50^a^	57.37^a^	0.004	0.037	0.002	0.004

DM, dry matter; CP, crude protein; EE, ether extract. *N* = 4.

### Serum inflammatory cytokines and immunoglobulins

As shown in Table 3, dietary PRO supplementation significantly decreased the serum concentrations of pro-inflammatory factors TNF-α (linear: *p* = 0.001; quadratic: *p* = 0.001) and IL-1β (linear: *p* = 0.023; quadratic: *p* = 0.001) as PRO levels increased from 0 to 120 mg/kg. Moreover, the concentrations of immunoglobulins such as IgA (linear: *p* = 0.001; quadratic: *p* = 0.001), IgG (linear: *p* = 0.002; quadratic: *p* = 0.001), and IgM (linear: *p* = 0.001; quadratic: *p* = 0.001) showed a linear and quadratic increase with increasing levels of PRO supplementation.

**Table 3:** Effect of PRO supplementation on serum inflammatory cytokines and immunoglobulins in the weaned pigs.

Items	PRO (mg/kg)					SEM	*p*-value		
	0	15	30	60	120		ANOVA	Linear	Quadratic
TNF-α (pg/mL)	105.40^a^	86.26^b^	82.41^b,c^	77.88^b,c^	70.73^c^	2.62	0.001	0.001	0.001
IL-1β (ng/L)	5.75^a^	3.87^b^	3.49^b^	3.76^b^	2.75^b^	0.31	0.023	0.001	0.005
IL-6 (ng/L)	107.92	104.64	115.12	113.56	120.08	3.37	0.621	0.131	0.325
IgA (μg/mL)	4.78^d^	5.37^c^	6.42^c^	9.30^b^	11.69^a^	0.54	0.001	0.001	0.001
IgG (μg/mL)	38.70^b^	52.38^a^	56.12^a^	58.99^a^	61.65^a^	2.12	0.001	0.002	0.001
IgM (μg/mL)	0.74^c^	1.30^b^	1.97^b^	2.88^b^	4.30^a^	0.27	0.002	0.001	0.001

TNF-α, tumor necrosis factor alpha; IL-1β, interleukin-1 beta; IL-6, interleukin 6; IgA, IgG, IgM, immunoglobulin A, G, M. *N* = 8.

### Serum antioxidant capacity

The effects of dietary PRO supplementation on serum antioxidant capacity in the weaned pigs are shown in Table 4. Compared to the CON group, the activity of GSH (linear: *p* = 0.048; quadratic: *p* = 0.016) and T-AOC (linear: *p* = 0.036; quadratic: *p* = 0.001) increased significantly with rising PRO contents, while the MDA concentration showed a linear decreasing trend (*p* = 0.056). In addition, dietary PRO supplementation significantly improved the. OH (linear: *p* = 0.016; quadratic: *p* = 0.041) and O^2−^ (linear: *p* = 0.001; quadratic: *p* = 0.001) scavenging activities, showing linear and quadratic increases with increasing levels of PRO inclusion in the diet.

**Table 4:** Effect of PRO supplementation on serum antioxidant capacity in the weaned pigs.

Items	PRO (mg/kg)					SEM	*p*-value		
	0	15	30	60	120		ANOVA	Linear	Quadratic
CAT (U/mL)	31.36	29.54	44.15	41.41	40.41	2.92	0.425	0.057	0.168
T-SOD (U/mL)	202.18	192.53	194.51	209.20	211.01	0.223	0.223	0.120	0.080
MDA (nmol/mL)	1.79^a^	1.41^a,b^	1.35^a,b^	1.25^a,b^	1.02^b^	0.11	0.274	0.056	0.146
GSH (U/mL)	590.86^b^	657.59^a,b^	656.32^a,b^	716.53^a^	627.83^b^	12.50	0.019	0.048	0.016
OH (U/mL)	683.33^b^	743.67^a,b^	772.48^a^	785.32^a^	800.83^a^	14.73	0.084	0.016	0.041
•O^2−^ (U/L)	211.90^c^	230.77^b,c^	247.18^a,b^	267.18^a^	281.94^a^	6.57	0.002	0.001	0.001
T-AOC (U/mL)	2.14^c^	3.20^b^	4.72^a^	3.81^a,b^	3.41^b^	0.21	0.001	0.036	0.001

CAT, catalase; MDA, malondialdehyde; GSH, glutathione; T-SOD, total superoxide dismutase; T-AOC, total antioxidant capacity; O^2−^, superoxide anion radical scavenging activity; OH^−^, hydroxyl radical scavenging activity. *N* = 8.

### Serum biochemical parameters

**Figure 1:**
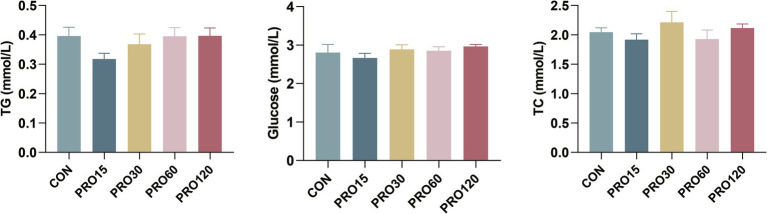
Effect of PRO supplementation on serum biochemical parameters. GLU, glucose; TG, triglyceride; TC, total cholesterol. CON: The pigs were fed a basal diet; PRO15, PRO30, PRO60, and PRO120 groups: The pigs were fed a diet containing 15, 30, 60, and 120 mg/kg of PRO. *N* = 8. Abbreviations and statistical notation are consistent across all figures and tables.

The effects of dietary PRO supplementation on serum biochemical parameters in the weaned pigs are shown in Figure 1. There was no difference in serum GLU, TG, and TC between the CON group and the PRO supplementation group (*p* > 0.10).

### Microbial metabolites in the rectum

**Figure 2:**
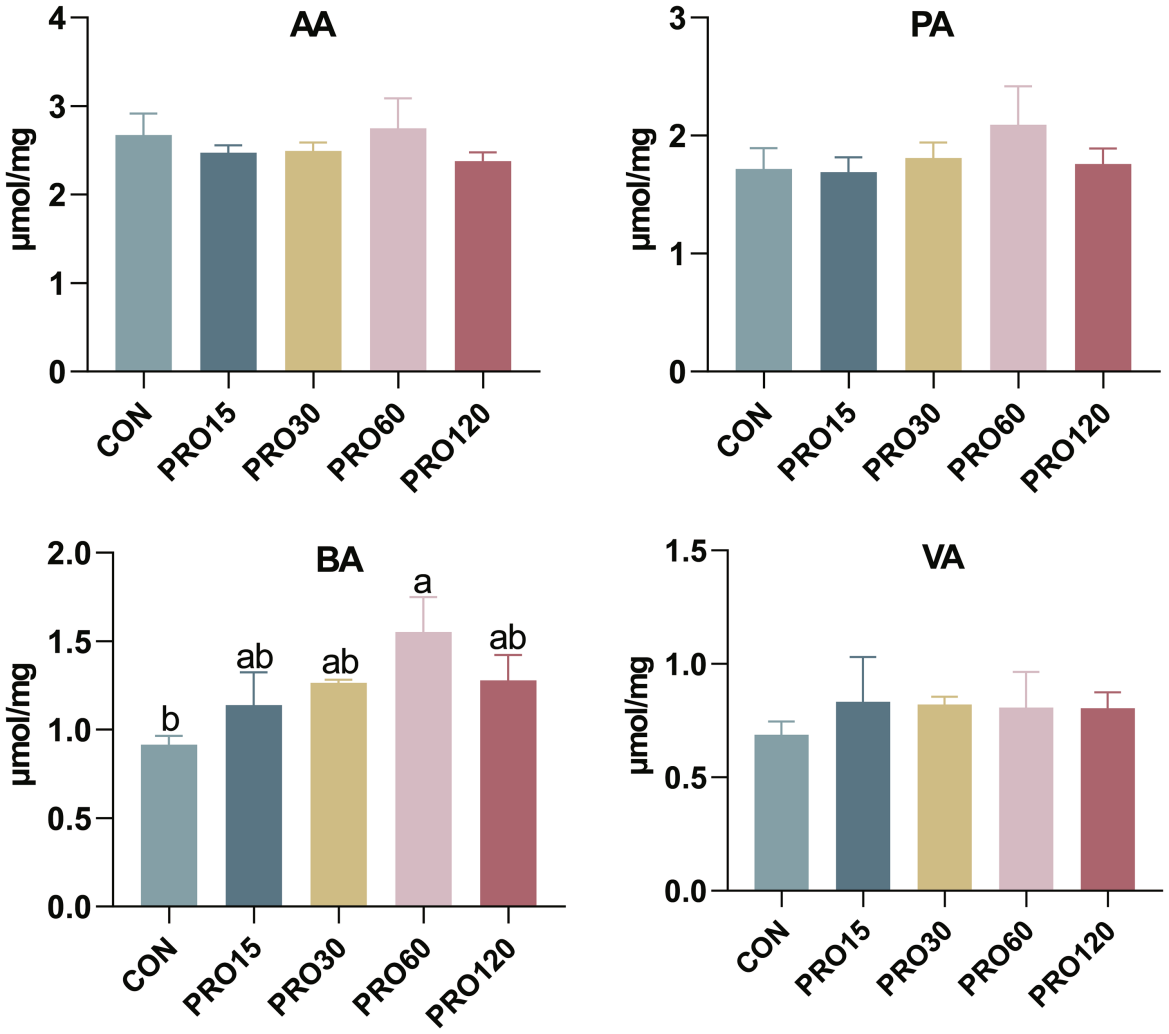
Effect of PRO supplementation on microbial metabolites in the rectum. AA, acetic acid; VA, valeric acid; PA, propanoic acid; BA, butyric acid. *N* = 4.

The effects of dietary PRO supplementation on microbial metabolites in the rectum are shown in Figure 2. Compared to the control group, supplementation with 60 mg/kg PRO significantly increased BA concentrations in the rectum of the weaned piglets, while other PRO supplementation groups showed a similar upward trend and a linear and quadratic increase with rising PRO levels (linear: *p* = 0.022, quadratic: *p* = 0.024).

### Gut microbiome diversity

**Figure 3:**
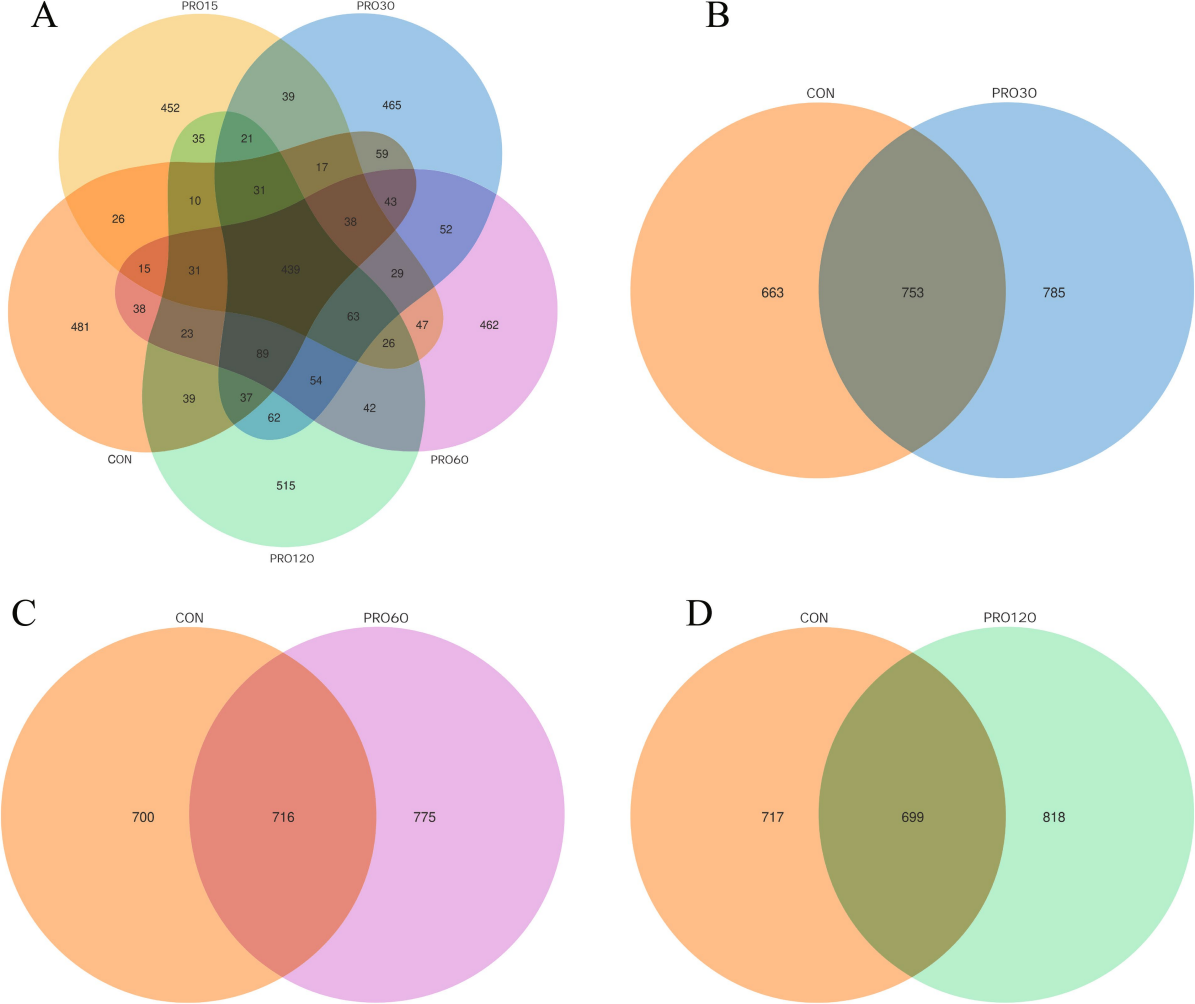
Venn diagrams showing the overlapping ASVs identified among the different treatments in the rectum. ASVs (amplicon sequence variants) represent the amplified sequence variants. **(A–D)** Venn diagrams showing the unique and shared OTUs among the different groups. *N* = 4.

After quality control, the DADA2 module or the deblur function in the QIIME2 software was used for noise reduction processing. Then, sequences with an abundance lower than 5 were removed, thereby obtaining the final ASVs, which represented the variant sequences that were amplified. As shown in Figure 3, 1,416, 1,319, 1,538, 1,491, and 1,517 ASVs were observed in the CON, PRO15, PRO30, PRO60, and PRO120 groups, respectively, and there were 439 identical ASVs across all five treatment groups.

**Figure 4:**
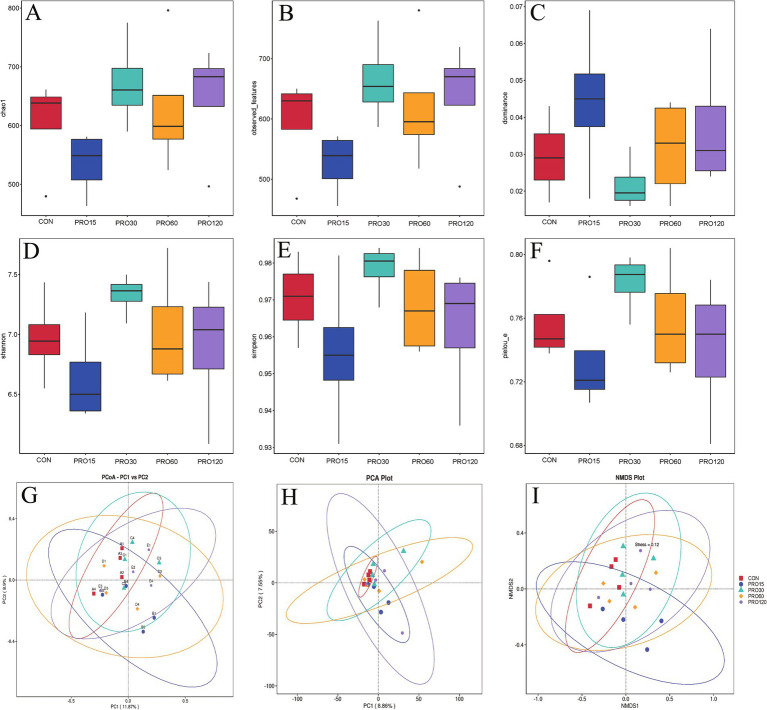
Comparison of microbial alpha diversity and beta diversity among the five treatments in the rectum. Alpha diversity indexes include Chao1 **(A)**, Observed_Features **(B)**, Dominance **(C)**, Shannon **(D)**, Simpson **(E)**, and PIEOU_e **(F)**. Beta diversity indexes include PCOA **(G)**, PCA **(H)**, and NMDS **(I)**. *N* = 4 for each group. *N* = 4.

To better understand the effects of dietary PRO supplementation on gut microbiota composition in the weaned pigs, five alpha diversity indexes (Observed_Features, Dominance, Shannon, Simpson, and PIEOU_e) were analyzed. However, no significant differences were found in alpha diversity among these five groups, suggesting that dietary PRO supplementation had no significant influence on the richness and diversity of the rectal microbiota in the pigs (*p* > 0.10) (Figure 4). In addition, PCoA and NMDS revealed significant differences in distances between the groups treated with varying PRO levels and the control group, suggesting that the effect of PRO on gut microbial composition may be related to PRO concentrations.

**Figure 5:**
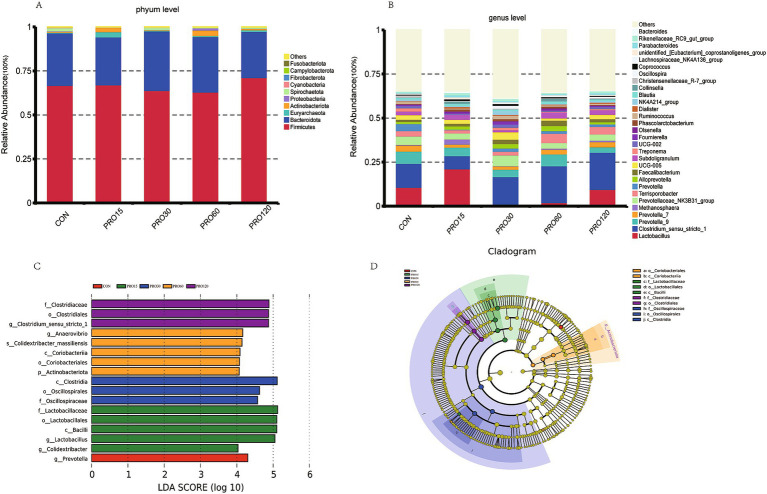
Effect of dietary PRO supplementation on microbial composition in the rectum. The relative abundance of microbes at the phylum level **(A)** and genus level **(B)**. LDA score **(C)** and cladogram **(D)** of microbes in different groups. *N* = 4.

The relative abundance of microbiota at the phylum and genus levels is shown in Figure 5. A total of 14 known phyla were recognized based on the quality testing results. Among them, Firmicutes (66.46%) and Bacteroidota (29.72%) were the predominant phyla identified in all rectum samples, but no significant differences were observed among the groups (*p* > 0.10). At the genus level, compared to the CON groups, 30 mg/kg PRO supplementation tended to increase the abundance of *UCG-005 and Fournierella* (0.05 < *p* < 0.1), significantly increased the abundance of *Oscillospira* and *NK4A214_group* (*p* < 0.05), and decreased the abundance of *Lactobacillus* (*p* < 0.05). Interestingly, the abundance of *Clostridium_sensu_stricto_1* showed linear and quadratic increases with increasing levels of PRO (linear: *p* = 0.009; quadratic: *p* = 0.028). The differences in rectal microbiota among the five groups were analyzed by LEfSe. The results showed that *g-Prevotella* was more abundant in the CON group, *Lactobacillus* and *Colidextribater* were enriched in the PRO15 group, *Anaerovibrio and Actinobacteriota* were enriched in the PRO60 group, and *Clostridium_sensu_stricto_1* was more abundant in the PRO120 group.

### The correlations between bacterial abundance and nutrient digestibility or SCFA contents

**Figure 6:**
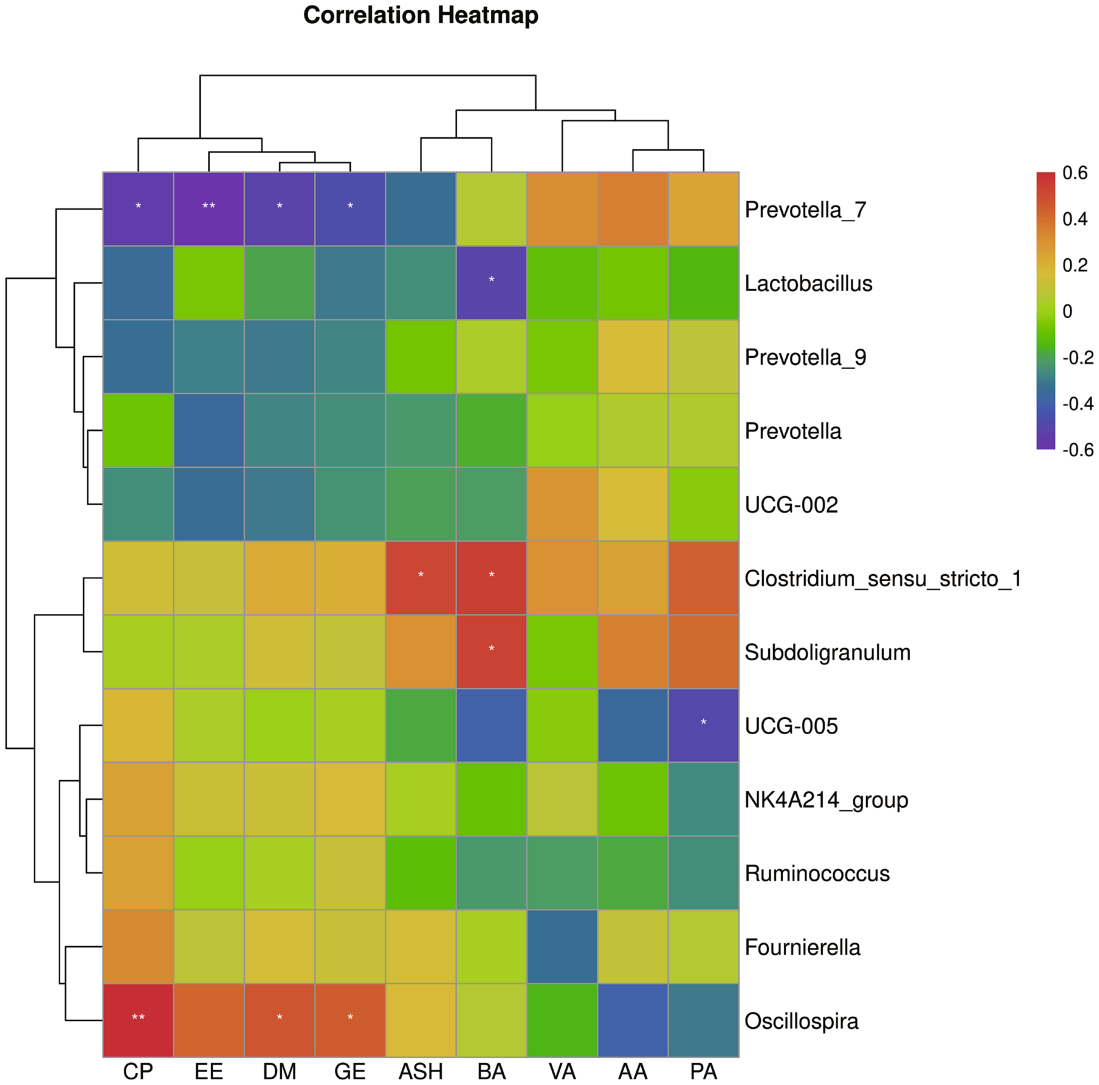
Correlations between bacterial abundance and nutrient digestibility or SCFA contents. Red indicates a positive correlation, and blue indicates a negative correlation. ^*^p < 0.05 and ^**^p < 0.01.

As shown in Figure 6, Spearman correlation analysis revealed significant associations between differentially abundant microbes among the top 30 taxa and nutrient digestibility, as well as short-chain fatty acids (SCFAs) concentrations. *Prevotella_7* exhibited a significant negative correlation with CP, EE, DM, and GE, whereas *Oscillospira* showed a significant positive correlation with CP, DM, and GE (*p* < 0.05). BA was positively correlated with the abundance of *Subdoligranulum* and *Clostridium_sensu_stricto_1* but negatively correlated with *Lactobacillus* (*p* < 0.05). In addition, *UCG-005* was negatively correlated with PA, while *Clostridium_sensu_stricto_1* was positively correlated with ash (*p* < 0.05).

## Discussion

As a class of commercially available polyphenolic compounds rich in hydroxyl groups, PRO possesses a wide spectrum of pharmacological functions, such as anti-tumor, anti-inflammatory, anti-oxidant, and anti-bacterial properties. Consequently, the inclusion of PRO in livestock and poultry diets may potentially confer beneficial effects on their overall health. For example, Yu et al. (2024) found that supplementation with 25–100 mg/kg PRO not only improved ADG and ADFI but also increased femur and tibia lengths in chicken. A study conducted on porcine epithelial cells revealed that PRO can mitigate the damage caused by co-infection with *E. coli* and *S. typhimurium* while reducing the adhesion rates of these two bacteria (Kovács et al., 2022). In this study, we observed increases in ADG and ADFI, accompanied by a marked improvement in nutrient digestibility, in the weaned pigs with rising PRO supplementation levels (15–120 mg/kg). These findings are consistent with previous studies (Zhang Y. et al., 2018; Song et al., 2011) reporting that dietary polyphenol supplementation extracted from plants could improve growth performance in animals. The increased production performance may be related to the improvement of intestinal barrier function and absorption capacity. Yan et al. (2021) discovered that PRO could alleviate acrylamide (ACR)-induced damage in Caco-2 cells by enhancing the expression of tight junction proteins and inhibiting the MAPK/MLCK signaling pathway. Moreover, several studies in common animal models (e.g., rats, fish, and chicks) have also found that PRO supplementation could promote digestion and absorption in the small intestine by improving intestinal morphology and nutrition transporter expression levels (Nyamambi et al., 2007; Skolness et al., 2011; Segú et al., 2022).

Immunoglobulins, a substantial Y-shaped glycoprotein molecule generated by plasma cells, serve as crucial components in the humoral immune system by specifically recognizing antigens, blocking their invasion, and enhancing the clearance of these antigens by phagocytes (Markina et al., 2020). IgG, the predominant antibody component in serum, is primarily synthesized and secreted by plasma cells in the spleen and lymph nodes, exerting an induced defensive effect against a wide range of bacteria, viruses, and fungi (Zhang T. et al., 2018). IgM is the first antibody type to be expressed in humoral immune responses, and its expression does not require processes such as class switch recombination (Li et al., 2020). In this study, the increased serum IgG, IgM, and IgA concentrations in the PRO groups demonstrated that PRO could improve the immunity of pigs. Moreover, TNF-α and IL-1β concentrations were maintained at lower levels under PRO supplementation compared to the CON group. TNF-α, IL-1β, and IL-6 are well-established markers of inflammation (Van and Bertrand, 2023). These findings are in line with multiple *in vitro* studies demonstrating that PRO has the capability to decrease the expression levels of iNOS, IL-6, and TNF-α by regulating the NF-κB and MAPK signaling pathways in IPEC-J2 or Caco-2 cells (Shi et al., 2023; Liu and Bolling, 2024). Moreover, a previous study also showed that PRO can alleviate LPS-induced inflammation by inhibiting macrophage polarization toward the M1 phenotype through the NF-κB signal pathway, thereby reducing the elevated levels of inflammatory cytokines. Based on the results, our study revealed that PRO supplementation enhanced the immune response of the piglets in large-scale breeding environments against antigenic invasion, likely due to its ability to stimulate immunoglobulin production and reduce inflammatory factor secretion.

During the weaning process in piglets, changes in the environment and diet can trigger weaning stress responses, which, in turn, lead to the generation of a large number of free radicals. Reactive oxygen species (ROS) are oxygen-containing molecules characterized by high redox potential, including superoxide anion radicals (O₂^−^), hydrogen peroxide (H₂O₂), and the highly toxic hydroxyl radicals (OH^−^) (Mailloux, 2015). Under normal conditions, ROS are maintained at low levels within the body, where they play a crucial role in cellular metabolism and differentiation (Cronstein et al., 1983; Williams et al., 1974). When the antioxidant defense system fails to neutralize excess ROS, oxidative stress occurs, resulting in molecular damage that impairs cellular and systemic functions and contributes to the development of various diseases (Li et al., 2024). The enzymatic antioxidant system of the body (e.g., GSH-PX and SOD) eliminates free radicals generated during metabolic activities, thereby protecting biomolecules from oxidative damage (Lei et al., 2016). PRO exhibits remarkable antioxidant properties and free radical scavenging activity due to its unique molecular structure, which contains a high content of phenolic hydroxyl groups (Jin et al., 2020; Shi et al., 2004). Furthermore, PRO regulates oxidative balance by targeting and inhibiting harmful free radical signaling pathways, activating antioxidant pathways (Luo et al., 2016; Yvonne et al., 2008; Su et al., 2018). In this study, dietary supplementation with PRO not only enhanced the serum scavenging ability for OH^−^ and O₂^−^ in the piglets but also improved serum antioxidant enzyme activities (T-AOC, GSH) and exhibited linear and quadratic growth trends with increasing PRO levels. Moreover, MDA, a marker of oxidative stress, significantly decreased with higher PRO doses. The results are consistent with previous studies on mice and intestinal epithelial cells, suggesting PRO protects against oxidative stress by enhancing free radical scavenging capacity and antioxidant enzyme activity.

The host and its gut microbiota maintain a dynamic equilibrium, which is influenced by various factors such as diet and the living environment, both of which can modulate microbial composition (Zeng et al., 2017). In the rectal microbiota, the dominant phyla Firmicutes and Bacteroidota were consistent across all groups, indicating that PRO supplementation did not significantly alter the overall microbiota structure. However, at the genus level, PRO supplementation resulted in notable shifts in specific microbial populations. Supplementation with 30 mg/kg PRO increased the relative abundance of *UCG-005* and *Fournierella* and significantly enriched *Oscillospira* and *NK4A214_group*, which are known to be involved in SCFA production, particularly butyrate. *Oscillospira*, a Gram-positive genus capable of fermenting complex polysaccharides, is predicted to produce butyrate through a butyrate kinase-dependent pathway (Konikoff and Gophna, 2016). Its abundance has been inversely associated with inflammatory diseases, and polyphenols derived from natural sources are suggested to promote host health, partially by enhancing *Oscillospira* proliferation (Keerqin et al., 2021; Pan et al., 2020; Zhao et al., 2021; Wang et al., 2018). For instance, polyphenols from millet husk and green tea significantly increase *Oscillospira* abundance while suppressing pro-inflammatory cytokine expression, enhancing the expression of intestinal tight junction proteins and improving antioxidant capacity (Wang et al., 2018; Khare et al., 2020; Wang et al., 2019). Similarly, *Fournierella*, a genus within the *Ruminococcaceae* family, contributes to host health through SCFA production, including acetate, butyrate, and propionate (Lv et al., 2022). Research on *NK4A214_group* and *UCG-005* is limited, but these genera are involved in fiber degradation and SCFA production (Wang et al., 2024; Xu et al., 2022). Conversely, the abundance of *Lactobacillus*, a genus associated with lactic acid production, was significantly reduced. These findings suggest that PRO supplementation selectively promotes certain beneficial microbes involved in SCFA production, particularly butyrate, while inhibiting those that favor lactate production. Notably, *Clostridium_sensu_stricto_1*, a key butyrate-producing genus, showed linear increases in relative abundance with higher PRO levels, further supporting the previous point (Lv et al., 2022).

The modulation of the gut microbiota by PRO was further supported by the analysis of microbial metabolites, particularly SCFAs. Butyrate concentrations in the rectum of the weaned piglets were significantly elevated in the 60 mg/kg PRO group, with other PRO supplementation groups showing a similar upward trend. The increase in butyrate production is particularly noteworthy, as it can directly serve as an energy source for intestinal epithelial cells and offers multiple functions, including promoting gut development, regulating microbiota, enhancing antioxidant capacity, and boosting immunity (Li et al., 2021; Zhang et al., 2021). Interestingly, Spearman correlation analysis revealed significant associations between specific microbial taxa and nutrient digestibility. For example, *Oscillospira* showed a positive correlation with CP, EE, DM, and GE. This suggests that *Oscillospira* may contribute to improved nutrient digestion and absorption, possibly through enhanced butyrate production, which could support intestinal epithelial function and nutrient assimilation. Furthermore, *Subdoligranulum* and *Clostridium_sensu_stricto_1*, both involved in butyrate synthesis, positively correlated with butyrate concentrations and improved nutrient digestibility (ash), while reduced *Lactobacillus* abundance was negatively correlated with butyrate (Lv et al., 2022; Radjabzadeh et al., 2022). The dynamic changes in the gut microbiota and their metabolites observed in this study may partially explain the improvement in nutrient digestibility, but the specific mechanisms underlying these effects warrant further investigation through targeted bacterial treatments and *in vitro* cultures.

## Conclusion

In conclusion, dietary supplementation with PRO has potential benefits for improving growth performance and nutrient utilization in piglets, likely by enhancing antioxidant capacity and improving immunity. In addition, supplementation with 60 mg/kg PRO changed the composition of the gut microbiota, which contributed to the regulation of microbial metabolites such as SCFAs. Therefore, PRO can be supplemented at a recommended dosage of 60 mg/kg in piglet diets.

## Data Availability

The data presented in this study are publicly available. The data can be found here: https://www.ncbi.nlm.nih.gov/, accession PRJNA1378523.
